# An Innovative Sequence-to-Structure-Based Approach to Drug Resistance Interpretation and Prediction: The Use of Molecular Interaction Fields to Detect HIV-1 Protease Binding-Site Dissimilarities

**DOI:** 10.3389/fchem.2020.00243

**Published:** 2020-04-29

**Authors:** Nuno G. Alves, Ana I. Mata, João P. Luís, Rui M. M. Brito, Carlos J. V. Simões

**Affiliations:** ^1^Department of Chemistry, Coimbra Chemistry Centre, University of Coimbra, Coimbra, Portugal; ^2^BSIM Therapeutics, Instituto Pedro Nunes, Coimbra, Portugal

**Keywords:** drug resistance prediction, Molecular Interaction Fields, *sequence-to-structure* algorithm, binding-site dissimilarities, HIV-1 protease

## Abstract

*In silico* methodologies have opened new avenues of research to understanding and predicting drug resistance, a pressing health issue that keeps rising at alarming pace. Sequence-based interpretation systems are routinely applied in clinical context in an attempt to predict mutation-based drug resistance and thus aid the choice of the most adequate antibiotic and antiviral therapy. An important limitation of approaches based on genotypic data exclusively is that mutations are not considered in the context of the three-dimensional (3D) structure of the target. Structure-based *in silico* methodologies are inherently more suitable to interpreting and predicting the impact of mutations on target-drug interactions, at the cost of higher computational and time demands when compared with sequence-based approaches. Herein, we present a fast, computationally inexpensive, *sequence-to-structure*-based approach to drug resistance prediction, which makes use of 3D protein structures encoded by input target sequences to draw binding-site comparisons with susceptible templates. Rather than performing atom-by-atom comparisons between input target and template structures, our workflow generates and compares Molecular Interaction Fields (MIFs) that map the areas of energetically favorable interactions between several chemical probe types and the target binding site. Quantitative, pairwise dissimilarity measurements between the target and the template binding sites are thus produced. The method is particularly suited to understanding changes to the 3D structure and the physicochemical environment introduced by mutations into the target binding site. Furthermore, the workflow relies exclusively on freeware, making it accessible to anyone. Using four datasets of known HIV-1 protease sequences as a case-study, we show that our approach is capable of correctly classifying resistant and susceptible sequences given as input. Guided by ROC curve analyses, we fined-tuned a dissimilarity threshold of classification that results in remarkable discriminatory performance (accuracy ≈ ROC AUC ≈ 0.99), illustrating the high potential of *sequence-to-structure*-, MIF-based approaches in the context of drug resistance prediction. We discuss the complementarity of the proposed methodology to existing prediction algorithms based on genotypic data. The present work represents a new step toward a more comprehensive and structurally-informed interpretation of the impact of genetic variability on the response to HIV-1 therapies.

## Introduction

Drug resistance is one of the greatest threats of the twenty first century. Fundamentally, the problem resides in the development and spread of resistance-conferring mechanisms among infectious pathogens such as viruses and other microbial targets (McKeegan et al., 2002). Importantly, the selection of random mutations stands out as one of the main mechanisms of acquiring resistance, particularly relevant in viruses which mutate at high frequencies. RNA viruses, for instance, have a mutation rate estimated at 10^−4^ per nucleotide per replication, while DNA viruses have a rate of 10^−8^ per nucleotide per replication (Vere Hodge and Field, 2011; Mason et al., 2018). The extreme variability and rapid mutational spectrum of viral genomes, ongoing viral replication, and prolonged drug exposure linked with the selection and widespread of new drug-resistant strains is still a matter of great concern and importance, particularly in immunocompromised populations (Strasfeld and Chou, 2010; Mason et al., 2018). While a limited number of antiviral drug classes are getting approved for human use, an increasing resistance to some of the most effective available antivirals for HIV/AIDS, herpes, influenza and hepatitis, is being observed. Furthermore, the unpredictability of viral evolution and drug resistance means that antiviral treatments remain costly to the health care systems and are still associated with a significant risk of mortality, particularly in low- and middle-income countries (Irwin et al., 2016). Hence, *a priori* understanding and prediction of resistance against drug targets is of paramount importance toward developing more effective and longer lasting treatment options and regimens.

Antiviral drug resistance has been extensively studied in the rapidly mutating human immunodeficiency virus (HIV). HIV-1, in particular, is one of the most studied virus and the increasingly affordable and accessible genotypic data from clinical HIV-1 strains, together with corresponding data on strain susceptibility or resistance toward several drugs, have sparked the development of several genotypic interpretation systems for prediction of phenotypic drug resistance and therapy response based on genotype (Bonet, 2015). Said systems include (a) rule-based algorithms, including the *Agence Nationale de Recherche sur le Sida* (ANRS) (Brun-Vézinet et al., 2003), the Stanford HIV Drug Resistance Database interface (HIVdb) (Tang et al., 2012), Rega (Van Laethem et al., 2002), and HIV-GRADE (Obermeier et al., 2012a), which heavily rely on the periodic update of mutation-resistance profile lists, and on the knowledge of expert panels; and (b) machine learning-based algorithms trained on large sets of genotype–phenotype pairs to predict the *in vitro* resistance to a specific drug, with renowned examples such as *geno2pheno* (Beerenwinkel et al., 2003) and SHIVA (Riemenschneider et al., 2016). These sequence-based methods are relatively fast and low cost, justifying their routine use to support medical decision in HIV pharmacotherapy (Vercauteren and Vandamme, 2006).

The most relevant computational predictors of antiviral drug resistance currently available share the shortcoming of being purely based on genotypic sequence data. By disregarding the three-dimensional structural context and enzymatic function of the mutated amino acid residues, these systems fail to capture the links between genetic viral mutations and the corresponding mutation-induced structural changes to the effector protein viral machinery (Cao et al., 2005; Weber and Harrison, 2016; Khalid and Sezerman, 2018). This means that such methods are limited in their predictive power and interpretability toward novel mutations and combinations of mutations that go beyond the information accessible for training, such as mutation patterns that are encountered in only a small number of patients.

In contrast, structure-based methods hold potential to help understanding and eventually predicting resistance mechanisms for previously unknown data, shedding light on the elusive link between novel mutations and drug resistance. This may be justified by the fact that such methods can take advantage of available structural information on protein-ligand complexes and structural modeling of point mutations in the protein structure (Hao et al., 2012). Reported examples of the use of structure-based methods include the application of molecular docking to predict resistance or susceptibility of HIV1-PR to different inhibitors (Jenwitheesuk and Samudrala, 2005; Toor et al., 2011), the use of molecular dynamics simulations to study the impact of mutations on enzyme dynamics, stability and binding affinity (Hou and Yu, 2007; Agniswamy et al., 2016; Sheik Amamuddy et al., 2018), and the use of computational mutation scanning protocols to extract insights on free energy and binding affinity changes resulting from active site and non-active site mutations (Hao et al., 2010). Even though these methods are constantly adding new pieces to the puzzle and opening opportunities in the understanding of drug resistance, they suffer from various drawbacks, such as being time-consuming and offering limited predictive accuracy. As a result of such limitations, the primary challenge facing structure-based drug resistance prediction is to achieve an acceptable balance between prediction accuracy and computational efficiency to become both reliable and fast tools to be used in clinic context (Hao et al., 2012). In fact, some of the most recent reports describe the use of machine learning strategies merging both sequence and structural data in attempt to achieve such balance (Masso and Vaisman, 2013; Yu et al., 2014; Khalid and Sezerman, 2018).

In this contribution, we describe a fast, computationally inexpensive, *sequence-to-structure*-based approach to the prediction of drug resistance. The proposed workflow makes use of an archetypal GRID-based method (Goodford, 1985) involving the generation and comparison of Molecular Interaction Fields (MIFs). MIFs may be defined as the spatial variation of interaction energies between a molecular target structure and selected types of chemical probes laid out on a three-dimensional (3D) grid (Cruciani, 2005). The broad range of applications of MIFs extends from ligand-based methodologies, e.g., 3D Quantitative Structure-Activity Relationships (3D-QSAR) models, drug metabolism and pharmacokinetics (DMPK) predictions and pharmacophore elucidation, all the way to structure-based drug design, including binding site detection and molecular docking (Artese et al., 2013). Within the context of viral drug resistance, MIFs hold potential in capturing subtle, mutation-induced, chemical perturbations within the binding site of resistant or susceptible viral structures, thus representing a promising approach to anticipating the impact of mutations on the response to antiviral drugs with atomistic detail.

HIV-1 protease (HIV1-PR) is one of the most characterized viral enzymes, with extensive structural, inhibitor, and mutation data available (Weber and Agniswamy, 2009). As of late 2019, the RCSB Protein Data Bank (RCSB PDB, 2000) ranks HIV-1 as the virus holding the highest number of available structures (2,586), majorly obtained through X-ray crystallography. Of these, the PDB returns 662 entities with at least 90% identity to the HIV1-PR subtype B *consensus* sequence from a BLAST sequence search (Stanford University, 1998a). The search by *consensus* sequences of other HIV-1 subtype B enzymes (Stanford University, 1998a) returns 586 structures for reverse transcriptase and 190 for integrase. With such amount of structural information available, we have built the framework of the present work using HIV1-PR as our first case-study. Commercially available HIV-1 protease inhibitors (PIs) are competitive peptidomimetics with a core structural scaffold that mimics the tetrahedral transition state of HIV1-PR substrate. Although these drugs are chemically distinct, their active conformations are superimposable, and generally establish the same pharmacophoric interactions with their target (Wlodawer and Erickson, 1993; King et al., 2004; Qiu and Liu, 2011; Nayak et al., 2019). Many mutations in HIV1-PR translate into changes in the structure and binding site physicochemical environment, thus affecting the affinity of PIs and representing a hurdle to achieving long-term viral suppression (Irwin et al., 2016; Pawar et al., 2019; Wensing et al., 2019). A quantitative analysis of HIV1-PR drug-resistant mutation frequency, with particular focus on the binding site, was performed using public sequence datasets to support the potential of a MIF-based approach to capturing mutation-induced active site dissimilarities. From this perspective, the workflow proposed here encompasses the use of a conservative structural modeling step for the generation of a HIV1-PR structure from its respective amino acid sequence, and a MIF-based structural alignment and chemical dissimilarity detection step comparing the input *sequence-structure* pair with a carefully selected naïve, susceptible template *sequence-structure* pair. We demonstrate that the quantification of such dissimilarity, depicting the extent of structural, physicochemical and pharmacophoric alterations introduced by mutations, allows for an accurate prediction of HIV1-PR's resistance to PIs.

Compared with previous approaches reported in the literature, and to the best of our knowledge, this work stands out as a first implementation of a fast, *sequence-to-structure*-based algorithm capable of discriminating susceptible and resistant HIV1-PR sequences. Considering that the problem of mutation-induced resistance cuts across virtually all infectious diseases, we believe the approach reported herein may be extended to a wide range of microbial targets besides HIV-1, thus helping rationalize and personalize the therapeutic decision-making process.

## Materials and Methods

**Figure 1:**
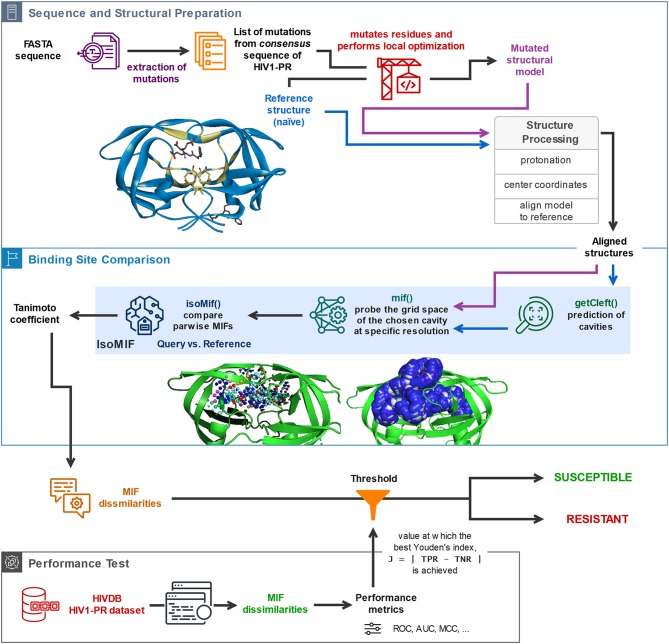
General description of the workflow underlying the proposed *sequence-to-structure*-based drug resistance classifier, holding platform applications to multiple microbial targets. The depicted algorithm starts off with the sequential reading of an amino acid sequence in FASTA format, followed by identification of the present mutations by comparison with the consensus sequence, insertion of the identified mutations in the naïve template, and processing of the structural models for alignment and comparison. Upon structural alignment of the target or database (predicted) structure with the naïve template structure, six types of MIF probe points are computed for the former structure and superimposed with pre-calculated MIF probe points of the latter structure. Calculation of MIF dissimilarities by means of a Tanimoto coefficient proceeds. The bottom panel represents the process of performance evaluation of the proposed classifier based on its application to a large dataset of *sequence-structure* pairs generated for HIV1-PR sequences retrieved from HIVDB. Included are performance metrics such as accuracy, Matthews Correlation Coefficient (MCC), and the area under the receiver-operating-characteristic curve (ROC AUC).

The availability of a public and curated database such as HIVDB (Stanford University, 1998c; Rhee et al., 2003) allows access to HIV1-PR sequences with known levels of resistance, and thus to establish datasets for the development of new methodologies to predict HIV1-PR resistance to protease inhibitors (PIs). This section describes the materials and methods employed in (1) the preparation of sequence datasets with various levels of resistance to PIs; (2) frequency analysis of *major* and *minor* mutations in the sequence datasets in (1); (3) the structural modeling of the reference structure used as template for subsequent modeling of HIV1-PR structures corresponding to each sequence in the datasets; (4) the core components of the proposed algorithm, including the calculation and comparison of pairwise Molecular Interaction Field points between the resulting structural models and the selected naïve template structure; and (5) the performance metrics used to test and evaluate the predictive power of the developed structure-based drug-resistance classification algorithm. A general workflow illustrating (4) and (5) is sketched (draw.io, 2005) in Figure 1 and the complete script *HIV1predict.sh* for running the sequences is available at GitHub (Alves et al., 2019b). Calculations were run on a 64-bit CentOS 6 Linux server with an Intel Xeon CPU (E5620) at 2.40 GHz (further information as Supplementary Table S1).

### Datasets of Resistant and Susceptible Sequences

A set of genotype-phenotype correlated HIV1-PR sequences was retrieved from HIVDB, version 8.7 (Stanford University, 1998b,c), and filtered by drug class for PIs. The considered PIs include darunavir, fosamprenavir, atazanavir, indinavir, lopinavir, nelfinavir, saquinavir, and tipranavir. Analyzing the subtype B HIV1-PR sequence of each isolate, i.e., a viral sample obtained from an infected individual, and considering positions with a mixture of amino acids, all possible mutation patterns were written to the FASTA format using a script written in-house (Alves et al., 2018f).

The genotype-phenotype correlation results from the *in vitro* PhenoSense assay (Zhang et al., 2005), which measures the levels of resistance to a PI compared to the wild-type sequence. Following the categorization of susceptibility to PIs described by Rhee et al. (2006), the collected sequences were classified as follows:

- *Susceptible*. Sequences holding <3.0-fold resistance to all PIs in the dataset were considered susceptible (*N* = 7,768) [Susceptible].
- *Resistant*. Sequences holding more than 20.0-, or 15.0-, or 10.0-fold resistance to all PIs, resulting in three resistant subgroups of increasing degree of resistance: respectively, [Res_20_] (*N* = 60) [Res20], [Res_15_] (*N* = 83) [Res15], which encompasses [Res_20_] plus 23 sequences holding between 15- and 20-fold resistance, and [Res_15_] (*N* = 873) [Res10], which encompasses [Res_20_] and [Res_15_] plus 790 additional sequences holding between 15- and 10-fold resistance.

#### Counting of Mutations in HIV1-PR

The quantification of *major* and *minor* mutations (Weber and Agniswamy, 2009) in all datasets was carried out using scripts written in-house (Alves et al., 2018a,b, 2019a) that sequentially read the listing of mutations for each sequence, extract either the *major* or *minor* mutations, and count them for each sequence. Said script was applied to quantify *major* and *minor* mutations in the HIV1-PR binding site.

### Preparation of HIV1-PR Structures

Using PDB's BLAST utility (Altschul et al., 1990) to guide the choice of a template for homology modeling, a sequence search, with a 10.0 E-value cut-off and at least 50% identity to the HIV1-PR subtype B *consensus* (Stanford University, 1998a), resulted in 784 entities available. With a more refined query of at least 95% identity to the HIV1-PR subtype B *consensus*, there were still 376 structures available to work with.

Out of these 376 structures, PDB entry 1NH0 for HIV1-PR was chosen as template structure for homology modeling by using PDB's BLAST utility (Altschul et al., 1990). It returned an E-value of 7.20281E-51, but since the intended work was heavily based on structure, our choice was also based on having the best resolution possible. The structure of 1NH0 holds 99% sequence identity (98/99) with the *consensus* B amino acid sequence of protease, HXB2 (henceforth referred to as *consensus sequence*), with one single mutation at position 37 (S37N), has 100% coverage of the sequence, and has been determined at 1.03 Å X-ray resolution. Importantly, this HIV1-PR sequence is known to be susceptible to all PIs.

In this work, Modeler version 9.19 (Šali and Blundell, 1993; Šali, 2019a) was used for predictive modeling of all HIV1-PR structures from their respective sequences. The listing of mutations present in each sequence was automated by scripting (Alves et al., 2018c) and followed by sequentially running the *mutate_model.py* script provided with Modeler (Šali, 2019b) to obtain the correct pattern of mutations and outputting the respective structural model. The procedure implemented in *mutate_model.py* performs local optimization of the mutated residues region and ensures that the obtained structural models are comparable to the template structure. The PDB structure itself (1NH0) was subjected to *mutate_model.py* in order to reverse the mutation present in the template with 99% identity (Asn37, on the outside of the protease) and keep on the *consensus sequence*, remove *HETATM* entries and *alt-locs*—thus yielding the reference template structure. This reference structure was used as template for the generation of the respective structural model of each input FASTA sequence present in the datasets.

All generated structural models were protonated using *Reduce*, version 3.23 (Word et al., 1999). The reference structure was centered to the origin of the axes of the cartesian coordinate system using *VMD*, version 1.9.3 (Humphrey et al., 1996). Structural alignment of all query models onto the centered reference structure was performed with *LovoAlign*, version 16.342 (Martínez et al., 2007).

### Workflow for Detection and Scoring of Molecular Interaction Field Dissimilarities

The MIF module of the software package IsoMIF, version dated March 2015 (Chartier and Najmanovich, 2015), was used to generate Molecular Interaction Fields (MIFs) within the HIV1-PR binding sites. MIF-based alignment and calculation of pairwise MIF dissimilarities between reference and dataset binding sites proceeded using the IsoMIF module of the same package. The IsoMIF setup comprises three sequential modules: GetCleft, MIF, and IsoMIF.

#### Cavity Detection (GetCleft Module)

GetCleft (Gaudreault et al., 2015) was employed to predict cavities in the structure of the reference HIV1-PR (Alves et al., 2018e). This geometry-based method detects cavities by insertion of spheres of radius *r* between the non-hydrogen protein atoms, reducing such radius if they intersect with any neighboring atoms (clefts defined by the union of overlapping spheres). First, the top five largest cavities were searched at the same time, with a minimum and maximum sphere radius of 1.5 and 4.0 Å, respectively. The largest predicted cavity was visually confirmed to be completely enclosed within the HIV1-PR binding site, using *VMD*, version 1.9.3 (Humphrey et al., 1996). Next, such cavity volume represented by spheres was used to define the location of MIF interaction vectors to be calculated for the reference and all 3D HIV1-PR structural models.

#### Generation of Molecular Interaction Field (MIF) Probe Points (MIF Module)

**Figure 2:**
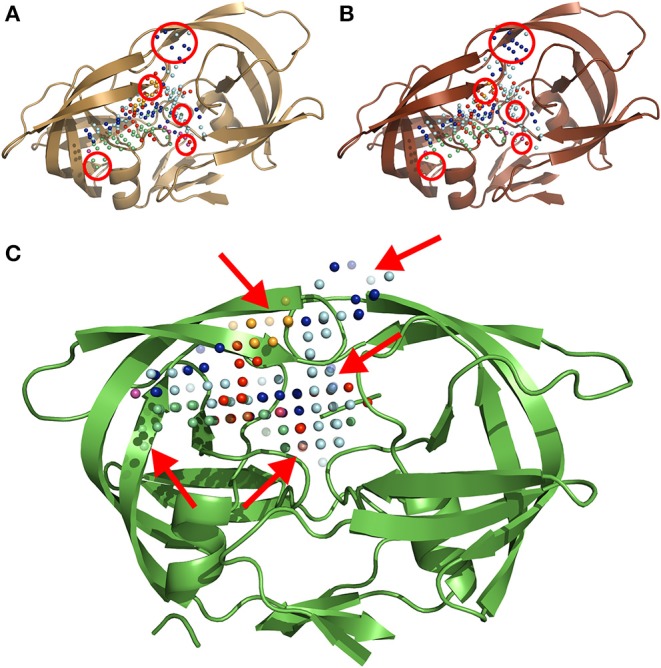
A three-dimensional ribbon depiction of the quaternary structure HIV1-PR dimeric unit, including the six different types of MIF probe points calculated on the enzyme's binding site. (A) Structure and probe points of the reference naïve template. (B) Illustration of the structure and probe points of a mutated model resulting from a sequence holding over 20-fold resistance for PIs. (C) An exemplary output of IsoMIF, highlighting a MIF-based alignment and comparison of both structures: (A,B). The red circles in (A,B) denote areas where the difference in probe points is most visible. The arrows in (C) point to semi-transparent probes, representing the probes which are not shared between the two structures. Legend for the six (6) probe types legend: hydrophobic in light blue, aromatic in orange, H-bond donor in dark blue and acceptor in red, positive in green and negative charge in purple.

The MIF module of IsoMIF was used to compute molecular interaction fields (MIFs) for six different chemical probe types (Figure 2): hydrophobic, aromatic, H-bond donor, H-bond acceptor, positive charge and negative charge. The pharmacophoric features shared by PIs (Wlodawer and Erickson, 1993; Nayak et al., 2019) highlight the importance of a conserved physicochemical environment in the binding site. Alterations of this environment are detected with the MIF probes (circled in Figures 2A,B) which allow for a quantification of changes caused by the presence of mutations. In this work, a grid resolution of 1.5 Å was defined to calculate the MIFs on the cleft covering the volume of the binding site. Such resolution was selected upon testing to achieve an adequate balance between speed and accuracy of IsoMIF pairwise field dissimilarity calculations.

#### Alignment of MIF Probe Points and Calculation of Dissimilarities (IsoMIF Module)

Field similarities were computed using the IsoMIF module, which employs a clique-based graph matching approach based on the Bron-Kerbosch algorithm (Bron and Kerbosch, 1973) to perform functional alignments between the probe points under comparison. A grid spacing of 1.5 Å, a geometric distance threshold of 1.0 Å and a maximum of 100 cliques were used as parameters for the calculation of similarities between the binding site of reference and structural models of HIV1-PR. Such similarities were then quantified by the Tanimoto coefficient (*T*_*c*__)_, calculated as in Equation 1:

(1) $$\begin{array}{l} T_{c}\;=\;\frac{N_{C}}{N_{R}+N_{Q}-N_{C}} \end{array}$$

where *N*_*c*_ is the number of common probe points to the two MIF maps under comparison; *N*_*r*_ and *N*_*q*_ represent the number of probe points present in the reference and query structure, respectively (Figure 2C) (Chartier and Najmanovich, 2015). The measurement of dissimilarity (Equation 2) between binding sites is justified by the fact that the focus of this work is set on the discrimination of resistant structures, when compared with a susceptible reference. Therefore, the chosen metric was dissimilarity rather than similarity:

(2) $$\begin{array}{l} dissimilarity\;coefficient\;=\;1.0-T_{c} \end{array}$$

### Analysis of Mutation Patterns Across Thousands of HIV1-PR Sequences

Analyses of the number and position of mutations were performed on HIV1-PR sequences in order to obtain information supporting and justifying the development of a *sequence-to-structure*-, MIF-based approach to antiviral resistance classification and prediction.

R version 3.4.3 (R Core Team, 2018) was used to conduct the analysis and generating the associated graphical representations. The R packages used in this work were ggplot2 (Wickham, 2009), *gplots* (Warnes et al., 2019), and ROCit (Khan and Brandenburger, 2019).

#### “Outlier” Detection on Binding-Site MIF Dissimilarities

Tukey's method (Tukey, 1949; Hoaglin, 2003), also referred to as Tukey's fences method, was used to detect outliers in the binding-site MIF dissimilarities results. Tukey's method is a statistical approach used to determine whether a value should be considered an outlier or not: the method relies on the interquartile range (IQR) measurement, which is calculated by the difference between the first quartile (Q1) and the third quartile (Q3) (see Equation 3). Q1 stands for the value in the dataset that holds 25% of the values below it and Q3 is the value in the dataset that holds 25% of the values above it.

(3) $$\begin{array}{l} IQR\;=\;Q3-Q1 \end{array}$$

According to Tukey's method, a value is considered an outlier if it is observed in the range described in Equation 4:

(4) $$\begin{array}{r} outlier<Q1-k\times IQR\vee outlier>Q3+k\times IQR\\ outlier<LowerBound\vee outlier>UpperBound \end{array}$$

where *k* = 1.5 indicates an outlier and *k* = 3 indicates an extreme outlier. For the purpose of the present work, only extreme outliers were discarded.

#### Evaluation of the Algorithm's Predictive Performance

The performance of our method at discriminating resistant from susceptible models was assessed by calculation of several metrics typically employed in the fields of predictive modeling and machine learning, particularly in cases where binary classification occurs. These included the Receiver Operating Characteristic (ROC) and the respective Area Under the Curve (ROC AUC). The ROC curve is a graphical representation of the True Positive Rate (TPR) as a function of the True Negative Rate (TNR), i.e., at various cut-off settings. The TPR is also known as Sensitivity (Equation 5), which measures the proportion of positive cases. On the other hand, the TNR is also calculated as 1—Specificity (Equation 6) and measures the proportion of true negative cases.

(5) $$\begin{array}{l} Sensitivity\;=\;\frac{TP}{TP+FN} \end{array}$$

(6) $$\begin{array}{l} Specificity\;=\;\frac{TN}{TN+FP} \end{array}$$

where *TP* represents the number of correctly identified resistant structures (true positives), *TN*, the number of correctly identified susceptible structures (true negatives), *FP*, the number of susceptible incorrectly predicted as resistant (false positives), and *FN* the number of resistant incorrectly predicted as susceptible (false negatives).

Additional performance metrics included Accuracy (Equation 7) and Matthews Correlation Coefficient (MCC; see Equation 8) (Matthews, 1975; Florkowski, 2008; Powers, 2011).

(7) $$\begin{array}{l} Accuracy=\frac{TP+TN}{TP+FP+TN+FN} \end{array}$$

(8) $$\begin{array}{l} MCC=\frac{TP\times TN-FP\times FN}{\sqrt{\left(TP+FP\right)\left(TP+FN\right)\left(TN+FP\right)\left(TN+FN\right)}} \end{array}$$

The dissimilarity threshold used for classification in resistant or susceptible *sequence-structure* pairs was derived from ROC curves, corresponding to the highest Youden's index (Youden, 1950), J, calculated as in Equation 9:

(9) $$\begin{array}{l} J\;=\;Sensitivity+Specificity-1 \end{array}$$

This index defines the maximum potential effectiveness of a classifier. It can be determined for all points of an ROC curve, although its maximum value represents the classifier optimal differentiating ability cut-point when equal weight is given to Sensitivity and Specificity (Ruopp et al., 2008).

## Results and Discussion

In this work, we describe a *sequence-to-structure-*, MIF-based method to assess binding-site dissimilarities across *sequence-structure* pairs, with the aim of predicting antiviral resistance—and using HIV1-PR as a case-study. It is generally accepted that the majority of resistance-conferring mutations occur in the binding site regions of viral enzymes (Weber and Agniswamy, 2009; Weber and Harrison, 2016). In order to further support the *rationale* and underlying assumptions of the proposed approach, we performed analysis of *major* and *minor* mutations of HIV1-PR binding site residues focusing on sequences known to be fully resistant and fully susceptible. For the sake of comparison, the quantification of mutations was also extended to *major* and *minor* mutations occurring in the remainder residues, i.e., residues not comprising the binding site region of HIV1-PR.

### Counting of PI-Resistant Mutations in HIV1-PR Sequences

Resistance to PIs develops upon accumulation of mutations that increasingly impact the structure of HIV1-PR, resulting in highly-resistant variants of HIV-1. As mentioned by Weber and Agniswamy (2009), PI resistance is linked to the occurrence of primary (*major*) mutations, commonly associated with the active site where HIV PIs typically bind, resulting from structural changes that disrupt the van der Waals contacts and/or hydrogen bonding patterns in the inhibitor-protein interaction and promote direct steric hindrance, by altering the pocket volume or its physicochemical environment. Secondary (*minor*) mutations occur in addition to *major* mutations, acting like accessory mutations that compensate the flaws produced by *major* mutations and enhancing the resistance level (synergistic effect). Being less obvious, they seem to affect HIV1-PR catalysis, dimer stability, inhibitor binding kinetics, and/or active site re-shaping through long-range structural perturbations (Weber and Agniswamy, 2009; Weber and Harrison, 2016).

Our workflow follows a *sequence-to-structure* approach in attempt to capture changes to the structural and physicochemical determinants of HIV1-PR's binding site upon mutation, based on the assumption that these changes represent the main driver of antiviral resistance. To support this assumption, quantification of mutations known to contribute to PI resistance was carried out across the retrieved datasets. The version 8.7 HIVDB (Stanford University, 1998b,c,d) listed the following PI-resistant mutations for HIV1-PR:

- *Major* mutations: D30N, V32I, L33F, M46IL, I47VA, G48VM, I50VL, I54VTALM, L76V, V82AFTSL, I84V, N88SD, and L90M;
- *Minor* mutations: L10FIVRY, V11IL, K20RIMTV, L23I, L24IFM, M36I, K43T, M46V, G48ASTQL, F53LY, I54ST, Q58E, A71VTIL, G73STCADV, T74PS, V82MC, N83DS, I84AC, I85V, N88TG, and L89VT.

**Figure 3:**
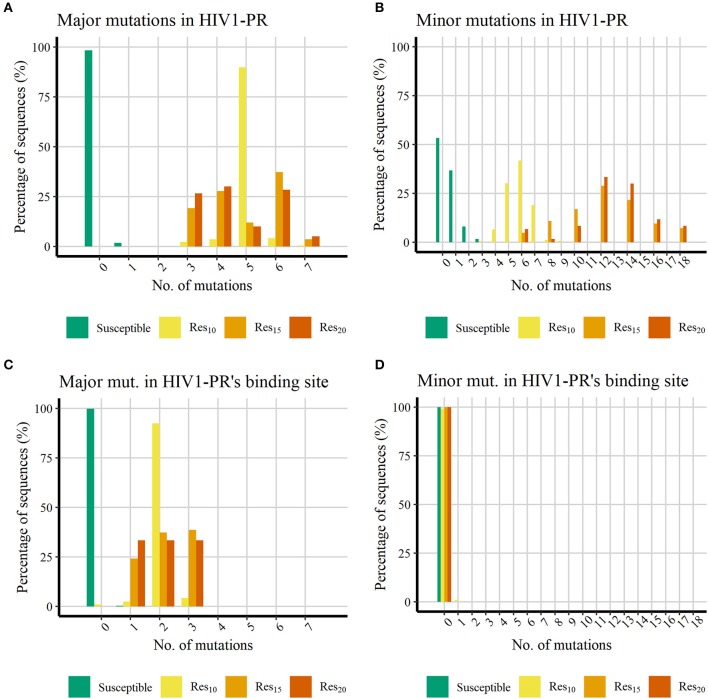
Histograms representing the percentage of PI-resistant mutations in the datasets showing increasing number of PI-resistant mutations in the datasets retrieved from HIVDB. (A) Percentage of major mutations in the whole HIV1-PR. (B) Percentage of minor mutations in the whole HIV1-PR. (C) Percentage of major mutations in the HIV1-PR binding site. (D) Percentage of minor mutations in the HIV1-PR binding site. Represented datasets: [Susceptible] (green); [Res_10_] (yellow); [Res_15_] (orange); [Res_20_] (dark orange).

Even though not all sequences exhibit the same degree of resistance to each PI, we selected these two groups of *major* and *minor* PI-resistant mutations and quantitatively characterized their presence in our subsets. Since all HIV1-PR sequences in our dataset were retrieved from the same unique source, HIVDB (Stanford University, 1998c; Rhee et al., 2003), the percentage of sequences holding PI-resistant mutations distributed across the entire HIV1-PR sequence, as well as the percentage of PI-resistant mutations manifesting in residues comprising the binding site of HIV1-PR, were determined and compared among all four subsets: [Susceptible], [Res_10_], [Res_20_], and [Resistant^*^]–as represented in Figure 3.

Figures 3A,B shows that, as expected, all HIV1-PR sequences belonging to the *Susceptible* subset hold much less PIs-resistant mutations than those belonging to the *Resistant* subsets. The majority (98.24%) of susceptible HIV1-PR sequences does not hold any *major* mutations, while 1.74% contain one *major* mutation, and only one sequence (0.01%) comprises three *major* mutations. The presence of *major* mutations across drug-resistant sequences is higher, ranging from three to seven *major* mutations, implying that among these subsets the *major* mutations appear in the shape of mutation patterns rather than individual mutations. The presence of *minor* mutations (Figure 3B) follows a similar trend to that witnessed for *major* mutations, with susceptible sequences denoting a lower number when compared to their resistant counterparts. Approximately 98.25% of the susceptible sequences present two or less *minor* mutations, with about half of susceptible HIV1-PR sequences (53.3 %) displaying no *minor* mutations.

When comparing susceptible vs. drug-resistant sequences, it can be observed that resistance against PIs is linked to the presence of *major* mutations, as implied above (Weber and Harrison, 2016). However, within the subsets of drug-resistant sequences, a direct relation between the number of *major* mutations and the increase of resistance is not observed. Drug-resistant sequences show a higher frequency of *minor* mutations, ranging from three to 18, with a visual apparent difference between sequences with lower resistance ([Res_10_]) and the more resistant sequences ([Res_15_] and [Res_20_]). In [Res_10_], 98.2% of the sequences have up to seven *minor* mutations, while 78.3% in [Res_15_] and 93.3% [Res_20_] have more than eight *minor* mutations. This trend in the profile of mutation distribution among the resistant sequences is in line with *minor* mutations acting as accessory mutations, appearing as patterns and not as individual mutations, and showing a similar trait as the one observed for the distribution of *major* mutations.

Analysis of *major* mutations located in HIV1-PR's binding site residues (Figure 3C), corresponding to sequence positions 30, 32, 47, 48, 50, 82, and 84, shows that 99.78% of the susceptible sequences do not display *major* mutations, while the remainder show only one *major* mutation. In contrast, less than 1% of resistant sequences lack *major* mutations in the drug binding site. Interestingly, the eight sequences representing this small fraction (0.91%) belong to the lower (10-fold) resistance subset ([Res_10_]). All remaining drug-resistant sequences hold from one to three *major* mutations in the enzyme's binding site.

Counting of mutations in binding site residues of HIV1-PR exposes a systematic presence of *major* mutations in resistant HIV1-PR sequences, while also highlighting the absence of such mutations on 99.78% of their susceptible counterparts. This contrasting trait observed between the binding site region of susceptible and resistant HIV1-PR supports the development of a structure-based drug-resistance classifier focusing on the detection and quantification of binding site dissimilarities.

Regarding the distribution of *minor* mutations across binding site residues, as represented in Figure 3D, mutations localized in sequence positions 23, 48, 82, and 84 were quantified among both HIV1-PR susceptible and drug-resistant sequences, revealing that the great majority does not present *minor* mutations in their respective binding sites. Only a small percentage of susceptible (0.01%) and resistant sequences (0.91%) show *minor* mutations in this region. It should be noted that the small subset of resistant sequences holding a *minor* mutation in their binding site region correspond to sequences that do not display *major* mutations in the active site.

These results show that the binding site *minor* mutations are uncommon on the datasets of HIV1-PR sequences—be they resistant or susceptible. Although such mutations appear to be important to increase the enzyme resistance's by stabilizing the mutated protein structure, they seem to produce limited direct effect on the enzyme's binding site, where they are mostly absent. Thus, these results seem to be in agreement with our motivation to explore a quantitative detection of binding-site dissimilarities to predict HIV1-PR resistance to PIs, as the *major* mutations play the main role on altering the binding site conformation, volume and/or physicochemical environment.

The quantification of mutations in the datasets retrieved from HIVDB yielded distinct results between the susceptible and drug-resistant sequences. Most of the resistant sequences show a higher frequency of *major* mutations when compared to the susceptible set. All resistant sequences present at least one mutation in the binding site region, contrasting with 98% of susceptible sequences that do not present any *major* mutations in that site. It is worth noticing that half of the *major* mutations are found in the binding site of resistant sequences. However, when considering the total number of mutations, the increase in the number of mutations per sequence seems to hold a reflection on the increase in the resistance of the observed sequence. Furthermore, binding site *major* mutations are more likely to cause changes on the HIV1-PR binding cleft physicochemical environment when compared with susceptible enzymes which do not have such type of mutations.

### A Fast, *Sequence-to-Structure-*, MIF-Based Antiviral Drug Resistance Classifier

The quantification of resistance-conferring mutations in HIV1-PR sequences, using the datasets retrieved from HIVDB, prompted us to further develop a discriminative resistance-classifier approach focused on analysis and comparison of binding-site MIFs. In practice, the proposed workflow involves performing structural modeling of input HIV1-PR sequences using the same template (i.e., 1NH0) and a script (Alves et al., 2018d) that calls *mutate_model.py* (Šali, 2019b) to conduct local energy minimization around the mutated residues of the HIV1-PR structure. Once the generation of structure models is concluded, the modules belonging to the IsoMIF package are deployed for cavity detection (GetCleft module), calculation of MIFs within the selected cavity volume (MIF module), field alignment and quantification of dissimilarities between MIF points computed for the dataset HIV1-PR structural models and those computed for a high quality [Susceptible] reference HIV1-PR structure (1NH0) and, finally, scoring by means of a Tanimoto coefficient (IsoMIF module). The average running time of the workflow is ≈ 77 s per sequence (Supplementary Figure S1 and Supplementary Datasheet S1), considering that this value varies with the amount of mutations present in the HIV1-PR.

### Analysis of MIF Dissimilarities in HIV1-PR Binding Site

**Figure 4:**
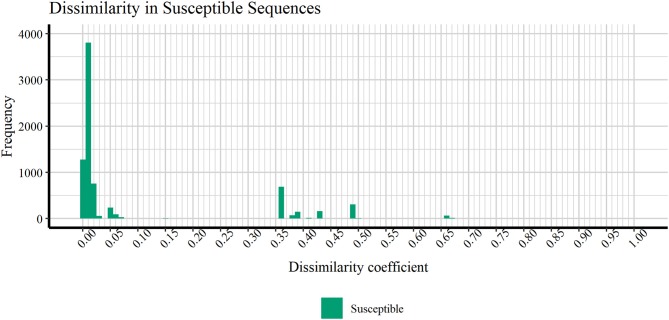
Frequency of susceptible sequences distributed across a range of HIV1-PR binding site 3D dissimilarity coefficients. The maximum value reported is ~0.667.

Figure 4 discloses the frequency of HIV1-PR *sequence-structure* pairs scattered across a spectrum of Tanimoto coefficient (Tc) values (varying from 0.00 to 1.00), in turn reflecting binding-site MIF dissimilarities in the subset of susceptible sequences (containing 7,768 *sequence-structure* pairs) against the selected naïve, template structure. Analyzing this profile of binding site dissimilarities, we observe that there are substantially more susceptible sequences concentrated on lower end of the dissimilarity spectrum. However, a small number of sequences (*N* = 81) present higher values, more visibly around the Tc value of 0.35. Since susceptible HIV1-PR *sequence-structure* pairs display a lower frequency of mutations in the binding site residues, we assume that Tc values deviating from the normal trend may highlight inconsistent data, errors and/or any form of outliers worthy of further investigation.

**Figure 5:**
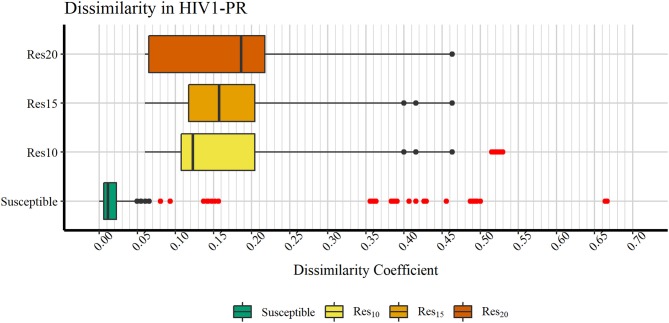
Boxplot representation of dissimilarity coefficients obtained for the HIVDB datasets. The maximum value reported is ~0.667. Outliers are marked in red. Represented datasets: [Susceptible] (green); [Res_10_] (yellow); [Res_15_] (orange); [Res_20_] (dark orange).

In order to verify if the higher Tc values could reflect true outliers, Tukey's outlier detection method was used (Tukey, 1949; Hoaglin, 2003). Table 1 shows the result of applying the statistical Tukey method to the MIF dissimilarity Tc values obtained for the dataset of susceptible *sequence-structure* pairs, and to the [Res_10_], [Res_15_], and [Res_20_] subsets. For each of the four groups, Figure 5 shows boxplots summarizing the distribution of the MIF dissimilarity Tc values. On the susceptible subset, the higher Tc values were identified as significantly different from the central tendency (values were below the determined lower bound; see Equation 4 in Methods). Looking at the dataset of resistant *sequence-structure* pairs, *extreme* outliers (as described in the Methods section) were only found in the [Res_10_] subset. These outliers were found to be associated with a software limitation wherein the same reference grid (generated by GetCleft), covering the entire binding site volume, was not homogeneous across all HIV1-PR structure models. In fact, a wider grid was calculated for some structures when compared to the reference HIV1-PR structure, which resulted on a different number of grid points, consequently leading to an increase of dissimilarities. Thus, these *sequence-structure* pairs were not considered relevant for performance evaluations, as they could introduce performance bias. The Tukey's boxplot analysis thus allowed the identification and removal of *extreme* outliers in the [Susceptible] and [Res_10_] subsets, resulting in 6269 and 680 HIV1-PR structural models, respectively. The [Res_15_] and [Res_20_] subsets remained unchanged with 83 and 60 HIV1-PR structural models, respectively. The resulting dataset has been used for further statistical analysis and as *test set* for performance calculations.

**Table 1:** Tukey's method results to determine outliers.

	***Susceptible***	***Res_**10**_***	***Res_**15**_***	***Res_**20**_***
Q1	0.0057	0.1075	0.1173	0.0649
Q3	0.0225	0.2041	0.2041	0.2171
IQR	0.0168	0.0966	0.0868	0.1522
Lower Bound	−0.0447^*^	−0.1823^*^	−0.1431^*^	−0.3917^*^
Upper Bound	0.0729	0.4939	0.4645	0.6737

*Quartile 1 (Q1), Quartile 3 (Q3), Inter Quartile Range (IQR), Upper Bound and Lower Bound values for susceptible sequences dissimilarity coefficient distribution. Upper and Lower Bound were calculated as described in Equation 4, with k = 3. Values above the upper bound and below the lower bound were considered outliers*.

* *Negative values are not realistic lower bounds; the minimum value must be 0*.

**Figure 6:**
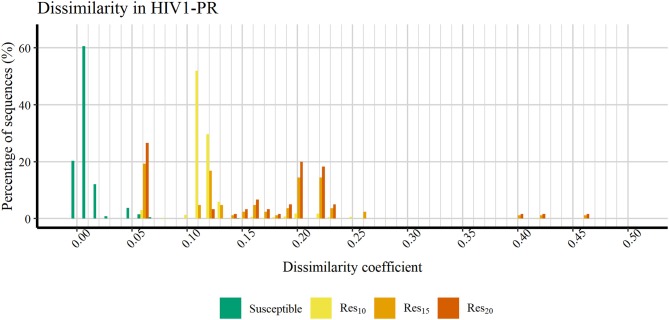
Percentage of sequences distributed across a range of HIV1-PR binding site 3D dissimilarity coefficients. Maximum value reported is ~0.463. Represented datasets: [Susceptible] (green); [Res_10_] (yellow); [Res_15_] (orange); [Res_20_] (dark orange).

Figure 6 shows a profile of the HIV1-PR binding-site MIF dissimilarities across the susceptible dataset withdrawn of extreme outliers ([Susceptible^*^]) and the stratified resistant data set (encompassing [Res_10_], [Res_15_], and [Res_20_]) also withdrawn of extreme outliers ([Susceptible^*^]). As seen, susceptible HIV1-PR structures tend to present very low to null binding-site MIF dissimilarities compared to the ([Susceptible]) structure modeled from the *consensus* sequence. In fact, 93.91% of the *sequence-structure* pairs in the susceptible group show dissimilarities lower than 0.02, indicating a considerable degree of conservation within the binding site. Overall, these results show a segregation between susceptible and resistant *sequence-structure* pairs, when analyzing their binding-site MIF dissimilarities against a susceptible reference sequence-structure pair, suggesting that our method is able to quantitatively capture differences among susceptible and resistant HIV1-PR structures.

### Evaluation of the Classification Performance of Our Drug Resistance Classifier

At the current stage of development, the proposed workflow only performs binary classification, meaning that each input sequence gets classified as either susceptible or resistant. Sequence data are used exclusively for the generation of the structural models on which dissimilarities are analyzed, but not to aid the classification itself. It is worth highlighting that our workflow relies on the detection of structural and chemical changes in viral enzymes that dictate susceptibility or resistance to drugs—rather than on the training of predictive models using sequences with known phenotypic response to drugs. Therefore, instead of using performance evaluation methods, such as cross-validation, that assess the impact of hiding a portion of training data (observations) on the accuracy of the resulting predictions, we resorted to the calculation of metrics of overall performance of our binary classifier.

The Receiver Operating Characteristic (ROC) curve was used to assess the overall discriminatory performance of our method. The score assigned to each dataset entry (here used for testing), corresponding to binding-site dissimilarities between each input *sequence-structure* pair and the template *consensus sequence*-structure, were thus plotted as a ROC curve. ROC curves are conceptually simple plots that depicts a binary classifier's discriminative capability as its discrimination threshold is varied. Such graphical plots are created by plotting the method's true positive rate (sensitivity) against its false positive rate (1-specificity), at varying thresholds. The area under the ROC curve (ROC AUC) value is a single scalar value varying between 0 and 1, providing a measure of the overall discriminatory power of the method. A ROC AUC value of 1 (or 100%) entails a *perfect* discrimination, a value of 0.5 represents random classification, while values above 0.8 are commonly accepted as indicators of an acceptable discriminatory performance (Fawcett, 2006; Pines and Everett, 2008; Powers, 2011; Tape). Furthermore, several performance measures, such as the Sensitivity (Equation 5), Specificity (Equation 6), Accuracy (Equation 7), and MCC (Equation 8) were also determined.

**Figure 7:**
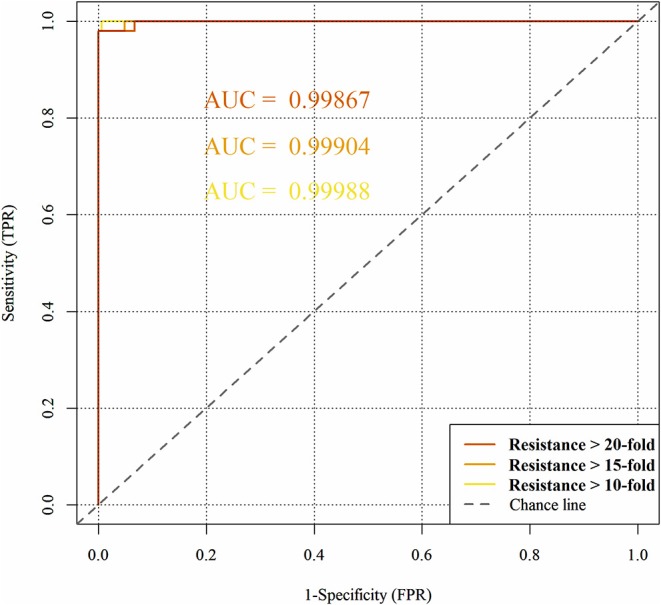
Predictive performance of the binary classification (resistant vs. susceptible) produced by the algorithm/workflow presented herein, quantified by means of Receiver Operating Characteristic (ROC) curves and their respective Area Under the Curve (ROC AUC). The colors represent the ROC curves as follows: yellow for HIV1-PR sequences associated with 10-fold resistance; orange for HIV1-PR sequences associated with 15-fold resistance; and dark orange for HIV1-PR sequences associated with 20-fold resistance.

Figure 7 represents the obtained ROC curves and their respective ROC AUC values for the susceptible and resistant HIV1-PR binding-site MIF dissimilarities. ROC AUC values for [Res_10_], [Res_15_], and [Res_20_] subsets were found to be similarly very high-−0.9999, 0.9990, and 0.9987, respectively – suggesting that the method holds significant discriminatory power to distinguishing susceptible from fully resistant HIV1-PR *sequence-structure* pairs—based on their binding-site MIF dissimilarities to the [Susceptible] reference *sequence-structure* pair.

We have also used ROC curve analysis to guide the definition of an *optimal* discrimination threshold based on Youden's index (Equation 9) (Youden, 1950). The optimal threshold observed corresponded to a 0.06 dissimilarity Tc for all [Res_10_], [Res_15_], and [Res_20_] subsets. Table 2 presents the values of each performance metric obtained for each subset, when applying a classification threshold of 0.06. At this classification cut-off, the specificities and sensitivities were found to be 0.997 and 0.994 for the [Res_10_] subset, 0.997 and 0.952 for the [Res_15_] subset and 0.997 and 0.933 for the [Res_20_] subset, respectively. In all cases, there is strong discriminative performance toward susceptibility or resistance—as it can be appreciated by the high accuracy values highlighted in Table 2. Nevertheless, the best results are found for the [Res_10_] subset, with an accuracy of about 0.997. On the other hand, the subsets with increasing degree of resistance, [Res_15_] and [Res_20_], show only slightly worst results concerning Sensitivity determined at a threshold of 0.06.

**Table 2:** Performance metrics obtained using a dissimilarity threshold of 0.0603.

**Dissimilarity Threshold = 0.0603**	***Res_**10**_***	***Res_**15**_***	***Res_**20**_***
ROC AUC	0.99988	0.99904	0.99867
Sensitivity	0.994118	0.951807	0.933333
Specificity	0.992184	0.992184	0.992184
Accuracy	0.99669	0.996379	0.996366
MCC	0.98151	0.874199	0.833085

The overall predictive performance of our method was also evaluated by the Matthews correlation coefficient (MCC) on the three resistant subsets, which summarizes the sensitivity and the specificity of a classification method within a unique value, also varying between 0 and 1. A higher value of MCC indicates that the method has a better discriminatory performance. For the [Res_10_], [Res_15_], and [Res_20_] groups, MCC values of 0.982, 0.874, and 0.833 were, respectively, obtained. Still, such performance metrics seems to highlight the clear potential of our MIF-based method to predict drug resistance, especially within the most populated [Res_10_] group (MCC value close to 1).

### Positioning and Differentiation vs. Sequence-Based, PI-Resistance Prediction Tools

More than a decade ago, Lengauer and Sing pointed out the lack of commonly agreed benchmark (or test) datasets to assess and compare the performance of different prediction methods (Lengauer and Sing, 2006). The amount of available information on matched HIV genotype–resistance phenotype has increased significantly over recent years, with HIVDB embodying an important role as a centralized data repository (Rhee et al., 2003). As expected, sequence-based methods can make use of as much information as available to train their predictions, resulting in that they become proficient at “predicting” the phenotypic response for the sequences they have been trained on. Only in a few cases do we witness a concern in drawing prospective validation on unseen sequence sets and in making those test sets available to the community (Tarasova et al., 2018). This hinders the design of fair comparisons with methods that do not make direct use of sequence data for training, such as the one we propose here. On the other hand, over the past years genotypic-based methods have reached a level of sophistication that allows them to perform resistance predictions to specific drugs, exclusively based on sequence data matched to phenotypic response, while, at its current stage of development, our MIF-based method can only perform binary classification (susceptible or resistant) of input sequences.

Taken together, these aspects render the comparison of our algorithm with existing, sequence-trained, multi-classification predictors *non-trivial* to say the least. Further developments of our methodology, aiming at a more exhaustive exploration of specific MIF areas around the mutated binding sites, may enable stratification of classification into multiple drug classes by detecting the determinants of resistance to specific PIs. For the time being, we center the analysis of differentiation of our method on the answer to a recurrent question in the mind virologists or physicians who prescribe HIV-1 medications: *would it be possible to accurately predict whether a new, unknown HIV-1 strain will be susceptible to known PIs?*

In order to answer to this question, we first converted our *test set* containing susceptible and resistant HIV1-PR sequences withdrawn of *extreme* outliers (*N* = 6,269 [[Susceptible^*^]] and *N* = 680 [[Resistant^*^]], respectively) into codon code, using the EMBOSS Backtranseq online tool (Madeira et al., 2019a,b), and then submitted it to the HIV-GRADE web server (Obermeier et al., 2012a,b) for comparison with the sequence-based algorithms ANRS-rules (Brun-Vézinet et al., 2003), HIVdb (Rhee et al., 2003; Tang et al., 2012) and Rega (Van Laethem et al., 2002; Camacho et al., 2017). Unexpectedly, we were not able to obtain predictions from *geno2pheno* via HIV Grade due to a technical issue of the web platform. To eschew this problem, we tried to submit the *test set* directly through *geno2pheno*'s web server, but the interface is limited to an unpractical maximum of 20 sequences per run.

Because the existing sequence-based interpretation systems try to predict phenotypical susceptibility or resistance to the individual drugs for a given genotype, whereas our approach only performs binary classification (susceptibility or resistance to all PIs), in order to draw comparison between the methods we tried to “level the playing field” by converting the predictions made by *sequence-based* algorithms into simpler binary classifications. In a first benchmark (benchmark A), the prediction outputs were converted into (i) susceptibility to all PIs ([Susceptible]) or (ii) resistance to any PI (*Resistant*). In a second, more challenging benchmark (benchmark B), the outputs were encoded as either (i) susceptible to all PIs (*Susceptible*) or (ii) resistant to all PIs (*Resistant*). The full list of criteria applied to the conversion of multiple classifiers into binary classification is given in Supplementary Table S2. The full raw output of HIV-GRADE is available in Supplementary Datasheet S2.

The ability to accurately predict the susceptibility of the input sequences to all PIs was assessed by determining the rate of correct predictions, with reflection into the calculated methods' Sensitivity (Equation 5) and Specificity (Equation 6). Table 3 lists calculated performance metrics for the *sequence-based* algorithms on both benchmarks A and B, contrasted with the performance of our *sequence-to-structure*-, MIF-based algorithm. Sensitivity_(A)_ and the number of detected false negatives FN_(A)_ translate the methods' ability to classifying a HIV1-PR sequence known to be resistant to all PIs as *Resistant to at least one PI*. In contrast, Sensitivity_(B)_ and FN_(B)_ translate the methods' ability to correctly predict the same sequences (known to be resistant to all PIs) as *resistant to all PIs*. From the methods' sensitivity viewpoint, the assessment of the results of both benchmarks A and B has been important to counterbalance the crudeness of the conversion of a multiple classifier of resistance toward specific PIs into a binary classification. Benchmark A clearly biases sensitivity in favor of a multi-classifier by considering any resistance prediction (in number or kind of PI) for sequences known to be resistant to all PIs as *correct*, whereas benchmark B offers a more stringent evaluation of sensitivity wherein only *resistant-to-all-PIs* predictions for the same set of fully resistant sequences are considered as *correct*.

**Table 3:** Performance metrics for exemplary sequence-based prediction tools tested against the datasets compiled in this work.

**PI-resistance predictor**	**Sensitivity_**(A)**_**	**Sensitivity_**(B)**_**	**Specificity**	**FN_**(A)**_^‡^**	**FN_**(B)**_^‡^**	**FP^‡^**
HIV-GRADE 07/2019	1.0000	0.1471	0.9809	0	580 *12*	120 *8*
ANRS 29_11/2018	1.0000	0.1868	0.8493	0	553 *14*	945 *61*
HIVdb 8.9.1	1.0000	1.0000	0.8818	0	0	741 *21*
Rega 10.0.0	1.0000	0.0838	0.9804	0	623 *7*	123 *10*
MIF-based Drug Resistance Classifier^†^	0.9941	0.9941	0.9922	4 *1*	4 *1*	49 *11*

† *The proposed MIF-based drug resistance classifier is shown in the last row for comparison purposes*.

‡ False negatives (FN) corresponds to the number of sequences belonging to the Resistant^*^ dataset (withdrawn of extreme outliers) that were predicted susceptible to all PIs. False positives (FP) corresponds to the number of sequences belonging to the Susceptible^*^ dataset (withdrawn of extreme outliers) that were predicted resistant to at least one PI. In italics are indicated the number of viral isolates to which the sequences misclassified as FP belong. Rules for sensitivity analysis in (1) benchmark A [Sensitivity_(A)_]: resistance to one or more PIs is considered a correct prediction; and (2) benchmark B [Sensitivity_(B)_]: resistance to all PIs is considered a correct prediction.

As expected the discriminatory power of the methods in benchmark A is in stark contrast with that calculated for benchmark B. Sensitivity_(A)_ suggests that *sequence-based* methods slightly outperform our *sequence-to-structure-*, MIF-based classifier, with 100% correct predictions of *Resistant* sequences vs. a Sensitivity_(A)_ value of 0.994 obtained by our method. By contrast, benchmark B shows a considerable drop in performance by *sequence-based* methods at correctly predicting HIV1-PR sequences resistant to all PIs—aside HIVdb, which retains a Sensitivity of 1.000.

The results in Table 3 indicate that our workflow outperforms all other algorithms at identifying sequences susceptible to all PIs, with a Specificity of approximately 0.992, while its sequence-based counterparts display Specificities ranging from approximately 0.849 to 0.981. Still, it is worth noting that the large number of FP from the other *sequence-based* methods mostly come from the same isolates, similarly as mentioned above for FN_(B)_. This fact highlights the advantage of accounting for structural information besides genotypic data. While MIFs allow searching for differences in the structural and physicochemical environment of proteins, which might not be significantly affected by mutations for similar amino acids, *sequence-based* approaches will consistently search for mutations at positions of interest and consistently assign them the same classification. At an early-stage of development, our workflow's performance is quite satisfactory, considering that the ability of correctly classifying a sequence as susceptible to all PIs is a highly relevant step at the beginning of antiretroviral therapy—where a false positive weights more on the flexibility of first-line therapy regimens and, consequently, quality of life of the patient.

## Conclusion and Future Perspectives

In recent years, the availability of data in the form of matched HIV genotype–resistance phenotype has expanded greatly, enabling further training of statistical learning methods relating genotype to different levels of phenotypic resistance and against specific drugs. However, in spite of the increased access to and routine sequencing of HIV's genome in many countries, as well as the constant evolution of machine learning (ML)-based techniques, HIV's high mutation rate (estimated in 3 × 10^−5^ per nucleotide per replication) will continue to pose significant challenges: not only in terms of the constant demand for curation of genotypic and phenotypic data to be fed into ML algorithms, but also from the viewpoint of the interpretability and translation of said data into knowledge to assist the design of novel anti-microbial agents. Therefore, the exploration of innovative structure-based *in silico* approaches to the prediction of drug resistance, focusing at the molecular interface that bridges to drug design, holds clear interest and appeal as alternative or complement to some of the most developed sequence-based statistical methods.

In this contribution, we propose a novel approach to drug resistance prediction, which captures structural and physicochemical modifications induced by mutations in the binding site of an extensively studied viral target, HIV1-PR. We demonstrate that, even at an early, proof-of-principle stage of development, our methodology can identify HIV1-PR *sequence-structure* pairs belonging to three levels of increasing resistance—with impressively high accuracy—thus anticipating, on a purely structural basis, whether a given HIV1-PR sequence will translate into phenotypic resistance or susceptibility to PIs. Since our *sequence-to-structure*-based classifier does not rely on *training* from genotypic data and only uses an individual input sequence to derive the corresponding viral enzyme structure and yield a prediction, its potential real-world value in supporting clinical decision is clearly relevant. Due to the fact that the proposed workflow produced predictions of complete drug susceptibility to the HIV1-PR datasets with high predictive accuracy, said results highlight this methodology as a potential valuable resource on clinical practice. Being able to use the clinical isolate sequence data to accurately predict susceptibility to known PIs, before starting a therapeutic regimen, is of paramount importance to allow the initiation of PI-based therapy with the less expensive 1st generation PIs, resulting in an economic benefit to the healthcare systems. Importantly, even though the method performs analysis on thousands of structural data points (atomic coordinates and MIF points), classification into *susceptible* or *resistant* takes place in a *couple-of-minutes* time scale.

It is worth emphasizing, nevertheless, that there is obvious room for methodological improvement and expansion. The upgrade to multi-classification functionality, where target structures known to be susceptible to specific inhibitors and drugs are used as template for structural modeling, is a critical milestone that will pave the way to predicting resistance to those specific anti-microbial agents. The growing amount of three-dimensional structural data on microbial target-inhibitor complexes, coupled with more elaborate use of sequence data, fuels our belief in that an improved *sequence-to-structure*-, MIF-based drug resistance classifier, will be able to combine the strengths and overcome the shortcomings of current approaches.

Claims of greatness must be backed by adequate validation designs. While the current version of our workflow does not allow drawing comprehensive and direct comparisons with more advanced sequence-based predictors of resistance to specific HIV1-PR inhibitors, further developments to our method will also be accompanied by the assembly and sharing of stratified benchmark sets of susceptible and resistant microbial target sequences—enabling fairer comparisons to be made both by ourselves and the scientific community.

As implied in our concluding words, a clear expectation around this work involves extending the application of our method to other targets, other than HIV1-PR, with inherent and multiple patterns of genetic variation. We realize, however, that this expectation may only be fulfilled if workable amounts of data are shared among the scientific community. Undoubtedly, one of the most critical aspects facing drug resistance prediction is the development of community-wide efforts to prepare and share useful datasets and tools to facilitate improvement and performance evaluation of existing and novel methodologies—which should be a clear priority for researchers working in the field. By basing its development on the use of freeware, our method is freely-available for non-commercial use.

To conclude, we see the results presented here as a promising example of the potential application of combined sequence- and structure-based *in silico* methods to achieve a more detailed interpretation and prediction of the impact of mutations in drug resistance. The ever-increasing emergence and widespread of drug-resistance calls in for the development of more efficient strategies to combat microbial threats in several fronts—be that in the drug discovery research setting or the clinical and medical therapeutic decision realm.

## Data Availability Statement

The datasets analyzed and scripts for this study can be found in the PI-resistance_Prediction GitHub [https://github.com/subject-am/PI-resistance_Prediction]. Raw data supporting the conclusions of this article will be made available by the authors, without undue reservation, to any qualified researcher.

## Author Contributions

CS and RB developed the idea for the present work and provided critical revisions. NA, AM, and JL contributed equally to its conception, literature search, and manuscript writing. All authors contributed to manuscript revision, read, and approved the submitted version.

## Conflict of Interest

CS and RB are cofounders of the company BSIM Therapeutics, however all work reported in this article was carried out at the University of Coimbra. The remaining authors declare that the research was conducted in the absence of any commercial or financial relationships that could be construed as a potential conflict of interest.
