# Supplementary material for: An Innovative Sequence-to-Structure-Based Approach to Drug Resistance Interpretation and Prediction: The Use of Molecular Interaction Fields to Detect HIV-1 Protease Binding-Site Dissimilarities
Source: Front Chem. 2020 Apr 29;8:243. doi: 10.3389/fchem.2020.00243 (PMC7202381; doi:10.3389/fchem.2020.00243)
Supplement: Supplementary file 1 [file Table_1.DOCX]

Supplementary Material

# Supplementary Data

Supplementary Table S1. Hardware information for the CentOS Linux server where all calculations were performed.

| **Architecture** | x86_64 |
| --- | --- |
| **CPU op-mode(s)** | 32-bit, 64-bit |
| **Byte Order** | Little Endian |
| **CPU(s)** | 8 |
| **Thread(s) per core** | 1 |
| **Core(s) per socket** | 4 |
| **Socket(s)** | 2 |
| **Vendor ID** | GenuineIntel |
| **Model name** | Intel(R) Xeon(R) CPU E5620 @ 2.40GHz |
| **Stepping** | 2 |
| **CPU MHz** | 2393.995 |

Supporting Datasheet S1. Run time data for the execution of the full *HIV1predict.sh* script on 1000 HIV1‑PR sequences (500 susceptible and 500 resistant).

**
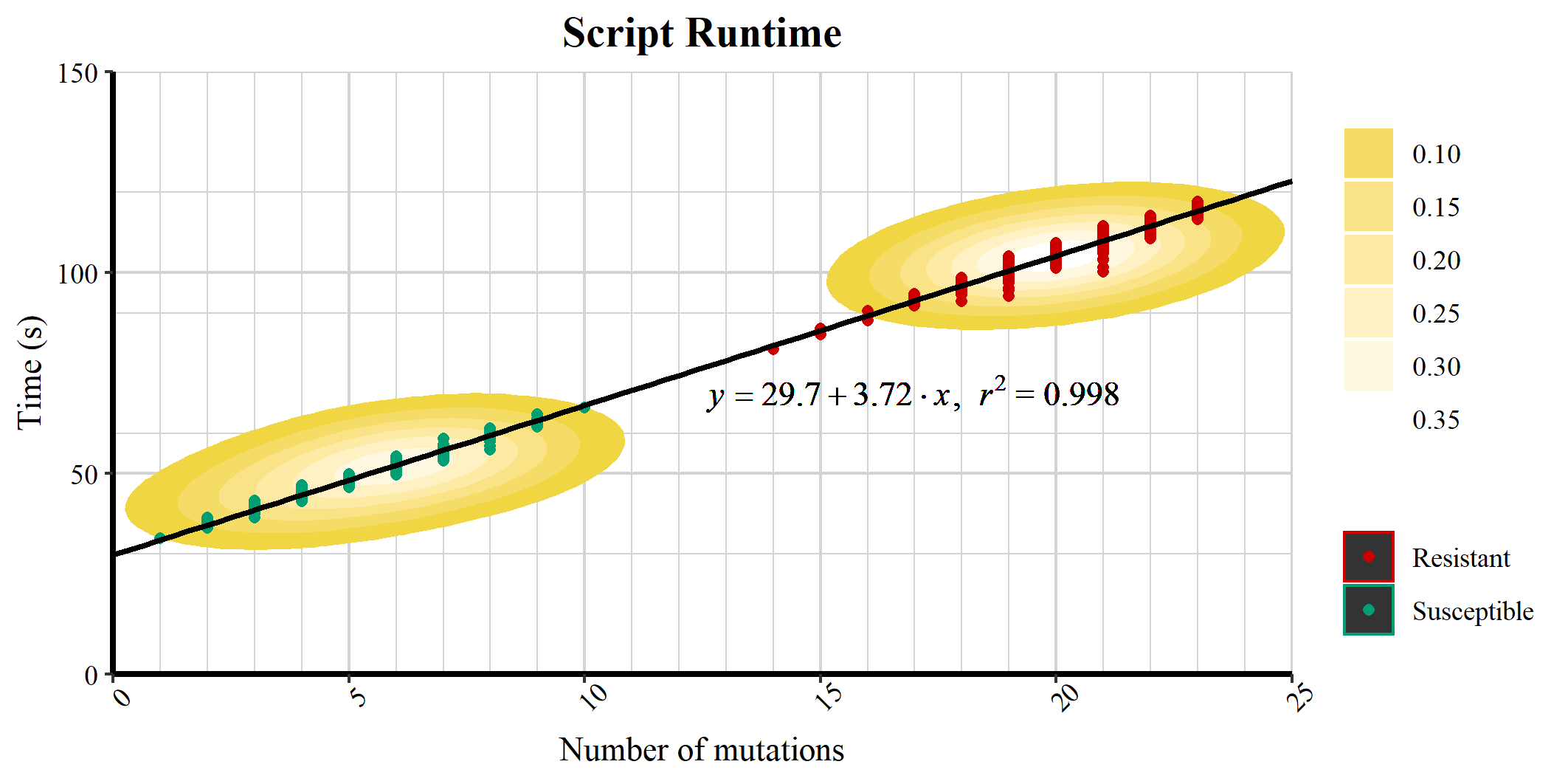
**

**Supplementary Figure S1.** Graphical representation of the CPU run time for the full *HIV1predict.sh* script (Alves, Mata, and Luís 2019b) as a function of the number of mutations (from the *consensus* HIV1‑PR sequence) of each sequence from a sample of 1000 sequences (500 susceptible and 500 resistant). In green are represented the susceptible sequences and in red are represented the resistant sequences. The density of sequences through the range of mutations are represented in different shades of yellow (low) to white (high). Figure and linear regression obtained from the R software, version 3.4.3 (R Core Team 2018).

Supporting Datasheet S2. Spreadsheet output from HIV-GRADE web server’s HIV-1 algorithm, for *Susceptible** and *Resistant** datasets used as test set.

Supplementary Table S2. Criteria applied to the conversion of multiple classifiers from existing *sequence‑based* prediction tools into a binary classifier ("Susceptible" or "Resistant") with the purpose of performance comparison with the sequence-to-structure‑, MIF‑based classifier proposed in this work.

|  | **Susceptible** | **Resistant** |
| --- | --- | --- |
| **HIV-GRADE 07/2019** | Susceptible; flagged mutations | Intermediate; Resistance |
| **ANRS 29_11/2018** | Susceptible | Possible resistance; Resistance |
| **HIVdb 8.9.1** | Susceptible; Potential Low-level Resistance | Low-level Resistance; Intermediate Resistance; High‑level Resistance |
| **Rega 10.0.0** | Susceptible GSS 1.5; Susceptible GSS 1 | Intermediate Resistant GSS 0.75; Intermediate Resistant GSS 0.5; Resistant GSS 0 |
